# A(H5N1) Virus Evolution in South East Asia

**DOI:** 10.3390/v1030335

**Published:** 2009-10-06

**Authors:** Ramona Alikiiteaga Gutiérrez, Monica Jane Naughtin, Srey Viseth Horm, Sorn San, Philippe Buchy

**Affiliations:** 1 Institut Pasteur du Cambodge, Virology Unit, 5 Monivong boulevard, PO Box 983, Phnom Penh, Cambodia; E-Mails: naughtin@pasteur-kh.org (M.J.N.); hsviseth@pasteur-kh.org (S.V.H.); 2 National Veterinary Research Institute (NaVRI)/ Phum Trea, Sals Street # 371, Phnom Penh, Cambodia; E-Mail: sorn.san@gmail.com (S.S.)

**Keywords:** avian influenza, H5N1 virus, evolution, South East Asia

## Abstract

Highly Pathogenic Avian Influenza (HPAI) H5N1 virus is an ongoing public health and socio-economic challenge, particularly in South East Asia. H5N1 is now endemic in poultry in many countries, and represents a major pandemic threat. Here, we describe the evolution of H5N1 virus in South East Asia, the reassortment events leading to high genetic diversity in the region, and factors responsible for virus spread. The virus has evolved with genetic variations affecting virulence, drug-resistance, and adaptation to new host species. The constant surveillance of these changes is of primary importance in the global efforts of the scientific community.

## Introduction

The Highly Pathogenic Avian Influenza (HPAI) H5N1 virus has dramatically affected human health and economies throughout South East Asia since its detection in 1997. Nearly half of the countries in Asia have declared H5N1 infections in humans. These account for 82% of the total number of confirmed cases, and 90% of fatalities worldwide [1,2]. The first H5N1 outbreak in poultry occurred in China in 1996 but the first human case was detected in Hong Kong in 1997 [3]. A large wave of HPAI H5N1 infections emerged in East and South East Asia in December 2003, rapidly affecting seven countries (Cambodia, China, Indonesia, Japan, Lao People’s Democratic Republic (Lao PDR), Thailand and Vietnam) [3–5] . The virus is now endemic in poultry in many of these countries and has caused repeated zoonotic infections in humans [2,6–8]. The virus has implications far beyond its impact on public health. H5N1 viruses have caused widespread disruption to poultry production and trade, particularly in South East Asia where a significant proportion of the population depends on farming for livelihood.

Like all influenza viruses, the HPAI H5N1 virus is able to rapidly evolve via mutations and reassortments of its segmented RNA genome. The prototype virus A/Goose/Guangdong/1/96 (A/Gs/Gd/1/96), which emerged in China in 1996, has undergone various genetic reassortments, and spread to neighboring countries in 2003. The direct precursor of the A/Gs/Gd/1/96 virus is unknown but is thought to be a Low Pathogenic Avian Influenza (LPAI) virus circulating in wild aquatic birds [9]. The probable progenitors that have contributed genetic elements to the A/Gs/Gd/1/96 lineage are probably H3N8 and H7N1 viruses from Nanchang (China), and H1N1 and H5N3 viruses from Hokkaido (Japan) [10].

In this review we examine the evolution of H5N1 viruses in South East Asia, the spatial and temporal transmission of H5N1 within South East Asia, and the emergence of clades and genotypes at a country level. Finally, we discuss significant genetic drift and shift mutations emerging in the H5N1 virus in South East Asia, and their significance in terms of drug-resistance, adaptation to different hosts’ species, and pandemic threat.

## History of emergence and circulation of H5N1 virus in South East Asian countries

### China

The Gs/Gd virus was initially isolated from geese in Guangdong Province, China in 1996. The Gs/Gd lineages are capable of zoonotic infections of human hosts via direct contact with infected birds. In Hong Kong SAR in 1997, a derivative of this strain caused the first fatal human case of H5N1 infection [11,12]. Southern China has often been proposed to be an epicenter for the generation of influenza pandemics, and indeed this region has continuously demonstrated the greatest H5N1 genetic diversity, suggesting that in addition of being the country of origin of H5N1 HPAI strains, Southern China is also an ongoing source of emergent and re-emergent HPAI viruses [13].

The circulation of over 20 different HPAI H5 and H7 subtypes has been documented in the last 100 years, however, only the Gs/Gd lineage has become so geographically widespread. Since 2000, HPAI H5N1 viruses have been repeatedly detected in Hong Kong SAR in live poultry markets and multiple novel genotypes have arisen through reassortment of Gs/Gd lineage viruses [4,14,15]. The analysis of 318 viruses isolated from 1996–2006 revealed that Gs/Gd lineage generated a total of 44 different reassortants in China [16].

The Hong Kong outbreak of 1997 was caused by a Hong Kong/156/97–like virus that derived its HA gene from Gs/Gd-like viruses and other genes from H9N2 or H6N1 viruses found in quail and other game birds sold in live markets [17,18]. During this outbreak, 18 humans were infected with H5N1 [2]. Six of these cases were fatal, giving a fatality rate of only 33%, which was much lower than subsequent H5N1 fatality rates. In response to the Hong Kong outbreak in 1997, millions of poultry livestock were culled, and as a result, a virus with the same gene constellation has not been detected again, indicating that the control measures used during this outbreak resulted in the eradication of this virus lineage.

H5N1 outbreaks re-emerged in poultry in 2003, and continued to spread throughout China in 2004, prompting a mass culling campaign to eradicate the virus. A total of 98 outbreaks in poultry were reported to the World Organization for Animal Health (or Office International des Epizooties, OIE) between 2003 and 2009 [19]. H5N1 has also been detected in wild birds in 2002 and during each winter season from 2004 to 2009 [5,20,21]. Outbreaks in poultry have again occurred in late 2008/early 2009 [5].

The H5N1 virus was not detected in humans again until February 2003 when two cases were reported in Hong Kong in a family who had recently travelled to Fujian province. Additionally, in November 2003, a human case was identified in Beijing [2]. Since late 2005, human cases have been reported from time to time in several Chinese provinces, and to date a total of 38 human cases have been confirmed in China, including 25 fatalities [2].

In 2005, hundreds of wild migratory birds became infected with H5N1, causing mass die-offs in Qinghai Lake in central China [22–25]. Since 2005, only two other outbreaks have been reported in Qinghai province [5]. In April 2006, the H5N1 virus was detected in wild birds, but did not significantly spread. Interestingly, the latest outbreak was reported on the 17th May, 2009 in Qinghai province, at Genggahu Lake. In this outbreak, 121 wild birds were found dead, and 600 backyard poultry were subsequently culled.

### Vietnam

H5N1 virus was introduced to Vietnam in late 2003 from Yunnan province, China, causing multiple poultry outbreaks and finally establishing itself endemically in this population [4,8,26] Outbreaks in poultry were first reported in 2004 and shortly followed by several human cases. Infections in humans were continuously reported throughout 2004–2005, with 65 cases reported during this period which at the time far outnumbered any other country [3]. Vaccination of poultry against H5 virus was initiated in Vietnam in August 2005, with inactivated H5N1 and H5N2 vaccines [27]. In efforts to control or eradicate H5N1 in poultry and to prevent human exposure, Vietnam has strengthened their vaccination campaign and associated surveillance programs in poultry, and improved poultry import controls and virological surveillance. Following the vaccination campaign, no influenza outbreaks were reported in poultry or humans from December 2005 until August 2006 [3]. The virus reappeared in poultry but no human cases were reported again until June 2007 and subsequently new human cases were regularly reported until the current time. At the time of writing, the total number of human infections detected in Vietnam was 111 with 56 mortalities, representing nearly one third of worldwide laboratory-confirmed human H5N1 infections [3,28]. Among 50 countries reporting H5N1 avian influenza in domestic poultry to the OIE, Vietnam has reported the greatest number, with 2539 outbreaks declared [5].

### Thailand

Avian influenza H5N1 virus was first reported in Thailand in January 2004 [29]. Several outbreaks were reported during the 2004–2005 period across many Thai provinces [29,30], prompting the government of Thailand to implement strict control and prevention measures against HPAI. The primary objectives were to identify geographic areas with confirmed H5N1 disease in poultry (e.g. 5-kilometer radius around an infected flock); to establish controls on the transport of poultry and poultry products out of affected areas, and to promote safe food-handling practices [29,31,32]. Small epidemics were reported in backyard flocks in 2006. However, following the tighter controls introduced during the same year, outbreak numbers and sizes were greatly reduced, leading to a much smaller natural reservoir which persists through the dry months [33]. Sporadic outbreaks continued to occur until the beginning of 2008 [34]. H5N1 transmission is seasonal in Thailand, as there have been several reports of backyard chicken deaths during the wet and cooler months of 2006/07 and 2007/08 [5,29,30,32–34].

Thailand has experienced 1141 outbreaks of H5N1 avian influenza in domestic poultry between 2003 and 2009 [19]. Approximately 63 million birds in three outbreaks were culled to prevent the further spread of the infection [30]. To date, 25 laboratory-confirmed human cases have been reported, including 17 fatalities [3]. The last human case of H5N1 was confirmed in September 2006 in a North-Eastern Thai province [3].

### Cambodia

In Cambodia the first confirmed outbreak of HPAI subtype H5N1 in poultry was reported during January 2004, and was followed by 14 other H5N1 outbreaks during the same year [35]. Interestingly, a retrospective investigation also revealed that between December 2003 through January 2004, a HPAI H5N1 outbreak occurred in a wildlife center in Takeo province, infecting several captive wild birds species, and cats. During the first four months of 2005, four fatal human H5N1 cases were detected in Kampot province, South East Cambodia, coinciding with deaths among poultry in this province [3]. In 2006, four outbreaks were detected in domestic poultry, and two human cases were reported [36,37]. In April 2007, Cambodia confirmed the seventh fatal human case of H5N1 infection from Kampong Cham province. The most recent case of human H5N1 infection (8th human case) was also the first non-fatal case and occurred in December 2008 in Kandal province. From 2004 to date, Cambodia officially reported 21 outbreaks of H5N1 avian influenza in domestic poultry [19]. The control strategies in poultry are based exclusively on culling of infected flocks, and the prohibition of poultry imports [35].

### Lao PDR

H5N1 avian influenza was detected in poultry in Lao PDR in 2003. A mass culling campaign was rapidly conducted, resulting in the loss of approximately 155,000 poultry. Surveillance of live bird markets during 2005 and 2006 failed to detect the virus, or serological evidence of exposure to the virus, suggesting that the virus from the initial outbreak had been eradicated [38]. In February 2006, avian influenza H5N1 virus was isolated from healthy ducks at a farm in Vientiane. Two human cases (both fatal) were reported in 2007 from Vientiane Province [2] In 2008 and early 2009, Lao PDR reported new poultry outbreaks of H5N1, however, no human cases were declared [3]. Since 2003, a total of 18 H5N1 outbreaks in poultry have been reported by Lao PDR authorities [19].

### Myanmar

Myanmar reported its first outbreak of H5N1 virus in poultry in March 2006, and subsequent outbreaks were reported in February, October, November, and December 2007 [3]. The sole human case of H5N1 infection (non-fatal) was reported in December 2007, in the Shan State province where an outbreak in domestic birds occurred at the same time. In March 2008, H5N1 sero-positive ducks were detected during routine surveillance conducted in the Shan State province [3]. To date, the country reported a total of 93 H5N1 avian influenza outbreaks in domestic poultry [19].

### Indonesia

Indonesia has suffered the heaviest burden of avian influenza, with 141 human infections, of which 115 were fatal [2]. Indonesia first detected H5N1 outbreaks in poultry in December 2003, and then continuously through to August 2006. H5N1 virus is now endemic in many Indonesian islands, such as Java, Sumatra and Sulawesi. To date, 261 outbreaks in poultry were reported in the country [19]. The first human case was reported in July 2005, and then human infections continued to occur frequently until the current time. Three family clusters of H5N1 infection were identified in 2005 [39]. In May 2006, Indonesia reported a large family cluster involving 7 family members and human-to-human transmission was suspected [40].

### Malaysia

Malaysia reported outbreaks its first outbreaks in poultry in August and September 2004. Additional outbreaks were reported in February and March 2006 in free-range poultry flocks and then again in chickens in June 2007. No human cases were detected in Malaysia [41]. Early detection and drastic culling measures could be attributed with such a successful control of H5N1 outbreaks in this country. A total of 16 H5N1 outbreaks in poultry were reported by Malaysian authorities [19].

### Comparison of fatality rates in patients from South East Asian countries

The 1997 H5N1 outbreak in humans in Hong Kong resulted in a fatality rate of 33%. Since the reemergence of H5N1 in 2003, this rate has been considerably increasing in most affected countries [2]. Of 429 confirmed human cases worldwide, 61% were fatal. South East Asia recorded 326 of these cases, including 222 fatalities, giving a fatality rate of 68% [2,42]. This rate is variable within each country. For example, China’s overall fatality rate is 66%, Indonesia’s 82%, Thailand’s 68%, Vietnam’s 50% (Table 1). The high fatality rate in Indonesia is striking, given the significant number of cases that have been reported there. Other South East Asian countries, such as Cambodia, Lao PDR, Myanmar, have small numbers of confirmed human cases (8, 2, 1, respectively), which means interpreting their fatality rates (88%, 100%, 0%, respectively) is difficult.

It is unclear whether H5N1 viruses in certain geographical regions differ in their pathogenicity. It would seem that clade 2.1 viruses (Indonesia) are more pathogenic than clade 1 viruses (Cambodia/Thailand/Vietnam) and 2.3 viruses (China) [43]. Drawing a link between H5N1 clade circulation and fatality rates is difficult, due to differences in health care practices and duration between onset of illness and treatment. Affordability of healthcare in developing countries and reduced access to anti-viral treatment at an early stage of the disease may partially explain increased fatality rates. Where surveillance is lacking, there is the possibility for under-representation of fatality rates, however, subclinical infections may also be under-estimated. Indeed, out of more than 600 blood-tested Cambodian villagers from areas where two children died in 2006, seven were seropositive for H5 antibodies indicative of asymptomatic infection. Thus, in this scenario the fatality rate was much lower than originally determined [44].

## Evolution of the Predominant Genotypes in South East Asia

South East Asian HPAI H5N1 viruses have their HA and NA genes derived from the prototype A/Gs/Gd/1/96 virus, whereas the genes encoding the six internal proteins (PB2, PB1, PA, NP, M, NS) are derived from several other sources. This diversity has allowed reassortment into various genotype groups, defined as unique gene constellations [4,14,16]. As for each of the internal gene segments constituting these constellations, a Neighbour-Joining bootstrap support above 70% or Bayesian posterior probability above 95% are the determinants for a distinct phylogenetic lineage [16]. However, in some cases, the same unique gene constellation has led to the definition of two genotypes, which only differ by some molecular marker. Genotypes Z and Z+, for example, differ by the presence or absence of a multi-amino-acid deletion on the NA protein [16].

**Figure 1.**
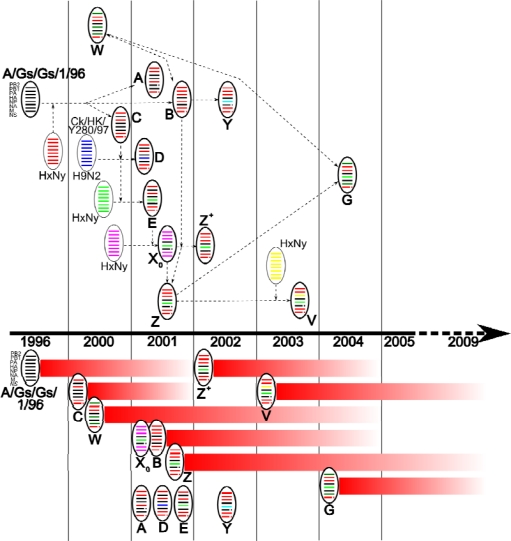
Diagram representing the emergence and persistence of major H5N1 reassortant viruses. Gene segments are ordered PB2, PB1, PA, HA, NP, NA, M, NS from top to bottom within the virus particle diagram. NS1 deletion (position 80–84) and NA-stalk deletion (position 49–68) are represented by discontinuous gene segments. Virus particles outlined in simple black represent potential donor viruses and those outlined in bold black represent characterized H5N1 genotypes placed at the year of first detection. Particles named “HxNy” represent potential donor viruses of which HA and NA subtypes were not identified. Arrows in dotted lines represent possible reassortment pathways of genotype development. Persistence of each genotype since first year of detection is represented by red lines, under the timeline.

By 2001, eight genotypes (A, B, C, D, E, X_0_, W, Z) had been identified in Hong-Kong and China (Figure 1), derived from A/Gs/Gd/1/96-like viruses, from an H9N2 avian virus (Chicken/Hong Kong/Y280/97), and other unknown viruses [4,14,16]. By 2003, three other major genotypes had arisen (Y, Z+, V) [4,16]. In 2004, the reassortment between genotypes Z and W led to the emergence of a new genotype, G [45]. Amongst all these genotypes, only a few persisted for longer than two years (B, Z, Z+, V, G, W, and X_0_) suggesting the acquisition of efficient survival capacities (Figure 1) [16].

These multiple reassortment events are believed to have occurred within domestic duck species, in which H5N1 generally causes an asymptomatic infection. It is proposed that avian influenza viruses from the natural gene pool existing in wild birds are introduced into domestic ducks, and are then able to reassort with endemic H5N1 viruses [45]. This would then enable the transmission of newly emerged reassortants to other poultry species, and facilitate their rapid spreading. Until recently, there was no evidence of reassortment events outside of China [16]. However, in 2008 and 2009, studies provided evidence of the emergence of local reassortants in Vietnam, Indonesia, and Thailand [28,33,45,46].

**Figure 2.**
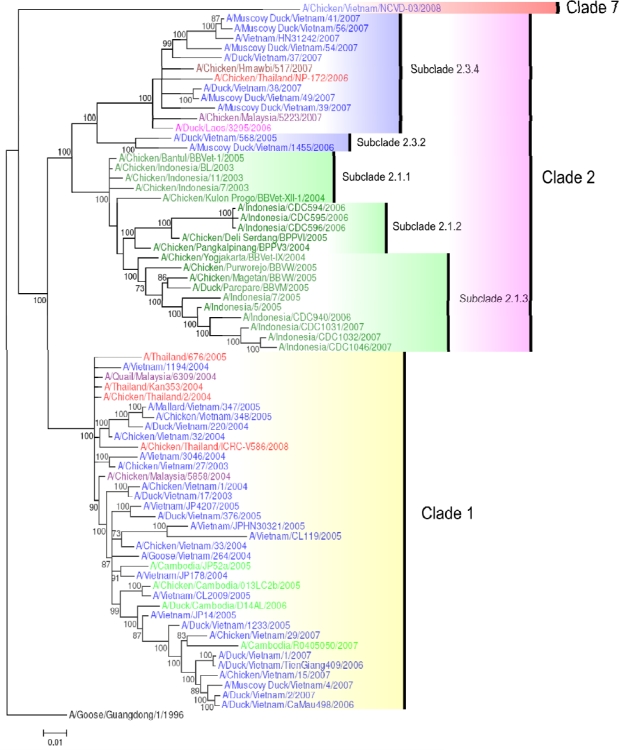
Phylogenetic tree of the HA gene of representative South East Asian H5N1 viruses. Analysis of HA gene based on full length gene sequences. The tree was generated by Bayesian analysis using MrBayes v3.1.2 [57]. Numbers on branches indicate Bayesian posterior probability values. The tree was rooted to A/Goose/Guangdong/1/1996. Scale bar: 0.01 substitutions per site. Colors used for the different countries are: Cambodia = light green; Indonesia = dark green; Lao PDR = pink; Malaysia = purple; Myanmar = maroon; Thailand = red; Vietnam = blue.

Genotype Z has dominated the H5N1 outbreaks documented in South East Asia from 2003 until the present day [47]. Nevertheless, in 2005 and 2006 several genotype G viruses were detected in poultry in Northern Vietnam, for example A/Duck/Vietnam/568/2005 and A/MuscovyDuck/Vietnam/1455/2006 [48] (Figure 2). These viruses may have been introduced into Vietnam in early 2005, since they were closely related to a virus first detected in Guangxi, China in that year [47]. Although seven clades and nine genotypes have been identified in Vietnam since first detection of H5N1 virus in the country [28,49], phylogenetic analysis of the internal genes reveal that at least 8 potential ancestors were necessary for the emergence of the viruses that circulated in this country, and subsequently provided a large pool of genes for further reassortments [28].

Although in Indonesia genotype Z is the sole genotype detected, there is now evidence of reassortment events within genotype Z viruses [46]. From 2003 to 2004, all detected viruses belong to a single phylogenetic group [46]. However, from 2005 to 2007, three groups were identified (named 1, 2, 3). Within group 2, subgroups of reassortant viruses were identified which were likely to have resulted from reassortment between group 3 ancestral viruses (M and PB1 genes), and group 2 ancestral viruses (PB2, PA, HA, NP, NA, NS genes). The common ancestor of these reassortant viruses has been dated to July 2005 [46].

All Thai strains of HPAI H5N1 viruses belong to genotype Z or Z^+^ with one exception which was a virus isolated in Nakhon Pathom province in 2006, A/Chicken/Thailand/NP-172/2006, which belongs to genotype V [50]. Although viruses from different clades have circulated in Thailand, no reassortment has been reported between clade 1 and 2 viruses. Nevertheless, important reassortment events occurred between various strains of H5N1 virus which persisted silently over a dry season period before giving rise to outbreaks in 2007–2008 [33].

In Lao PDR, the first H5N1 isolates detected in 2003 were genotype Z viruses. However, in 2007, a genotype V strain was isolated, and interestingly clustered with the genotype V A/Chicken/Thailand/NP-172/2006 virus, suggesting a common origin [51]. Suprisingly, only genotype Z clade 1 viruses have been detected in Cambodia since the emergence of the epidemic in 2003 [52].

## H5N1 Clade Evolution in South East Asia

Ten different clades (genetic groups) have been defined within the H5N1 viruses, based upon the evolution of the H5 hemagglutinin gene. These clades are strictly defined by phylogenetic criteria, such as sharing a common node, reaching a threshold value of ≥ 60 for the bootstrap at this clade-defining node and fitting in a precise range of values for the average percentage pairwise nucleotide distances between and within clades of >1.5% and <1.5%, respectively [53]. Several sublineages have emerged within the different clades, resulting in the designation of subclades.

Between 2003 and 2006, only clades 1 and 2 viruses circulated in South East Asia (Figure 2). Clade 1 viruses have circulated in Thailand, Vietnam, Malaysia, Lao PDR and Cambodia, affecting both poultry and humans. Clade 2 viruses in South East Asia are represented by subclades 2.1 (2.1.1, 2.1.2, 2.1.3) and 2.3 (2.3.2, 2.3.4). Clade 2 viruses have been circulating in Indonesia since 2003. Within Indonesian clade 2 viruses, subclade 2.1.1 viruses have caused outbreaks exclusively in poultry whereas subclades 2.1.2 and 2.1.3 have affected both birds and humans. Clade 2 (subclade 2.3.2) viruses emerged in Vietnam in 2005, infecting poultry and other migratory species. Subclade 2.3.4 viruses then emerged in 2006 and were identified in poultry outbreaks in Lao PDR, Vietnam (mainly in the north), Thailand, and Malaysia but never in Cambodia (Figure 2) [28,54,55]. These viruses were confirmed in human hosts in Vietnam for the first time in 2007 [56].

**Figure 3.**
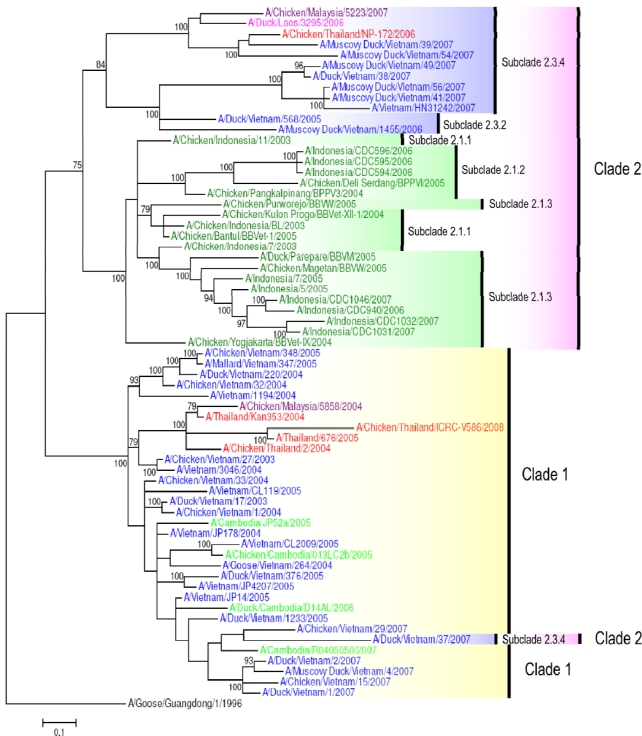
Phylogenetic tree of the NA gene of representative South East Asian H5N1 viruses. Analysis of NA gene based on full length gene sequences. The tree was generated by Bayesian analysis using MrBayes v3.1.2 [57]. Numbers on branches indicate Bayesian posterior probability values. The tree was rooted to A/Goose/Guangdong/1/1996. Scale bar: 0.1 substitutions per site. Colors used for the different countries are: Cambodia = light green; Indonesia = dark green; Lao PDR = pink; Malaysia = purple; Myanmar = maroon; Thailand = red; Vietnam = blue.

In Vietnam, seven clades and/or subclades have been identified: clades 0, 1, 2 (2.3.2, 2.3.4), 3, 5 [28], and recently clade 7 [49]. Viruses from clades 0, 3 and 5 persisted no longer than one to two years each [28]. Clade 7 viruses were detected in Northern Vietnam in 2008 (e.g., A/Chicken/Vietnam/NCVD-03/2008) (Figure 2). Subclade 2.3.2 viruses isolated in Vietnam are represented on the HA tree by the strains A/Duck/Vietnam/568/2005 and A/Muscovy Duck/Vietnam/1455/2006 (Figure 2), which are also the representatives for genotype G viruses (Figure 1). It is of interest to note that some viruses, such as A/Duck/Vietnam/37/2007, belong to the subclade 2.3.4, whilst sharing the NA and internal gene constellation of clade 1 viruses, providing evidence for mechanisms of reassortment between different clades (Figure 2 and 3) [48].

## Geographical Dynamics of H5N1 Virus Transmission

The H5N1 influenza virus has continued to spread from its established source in Southern China to other regions through the transport of poultry and bird migration [47]. Domestic ducks in Southern China have had a central role in the generation and maintenance of this virus, and wild birds may have contributed to the increasingly wide spread of the virus in South East Asia, and to other parts of the world [4,58,59].

**Figure 4.**
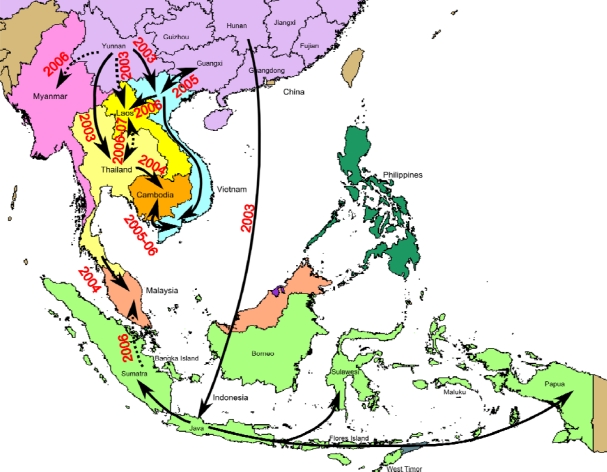
Map of major H5N1 migration events in South East Asia, based on epidemiological evidence. Arrows represent probable transmission routes. Arrows in dotted lines represent migrations for which the exact origin or the direction has not been elucidated.

Since 2003, the HPAI H5N1 virus has spread from China to other countries during three successive transmission waves in 2003, 2005, and 2006 [4,60,61]. The first wave of H5N1 outbreaks occurred in 2003/2004. These viruses originated in Yunnan and Hunan and became endemic in South Vietnam and Cambodia (clade 1 viruses), and Indonesia (clade 2.1 viruses), respectively (Figure 2 and Figure 4) [26]. The second wave of transmission occurred following the outbreak at Qinghai lake in Northern China in 2005, whereby clade 2.2 viruses were transmitted by migratory birds to Africa and Europe [22–25]. In the third wave, a Fujian-like sublineage (clade 2.3) replaced previously established sublineages in several provinces of China, before spreading to Vietnam, Thailand, Lao PDR, Malaysia and Myanmar in 2006 [61]. The event currently occurring in Qinghai province may be of some concern, since it is known to be on a major migration route of wild birds and of primary epidemiological importance.

The H5N1 virus has been introduced into Vietnam from Southern China on multiple occasions. The majority of novel viruses were first detected in Northern Vietnam, suggesting introduction from Yunnan (China) (Figure 4). These viruses subsequently spread to Southern Vietnam, often after reassorting with pre-existing local viruses in Northern Vietnam [26,28]. The apparent northern to southern spread of H5N1 may correspond to direct poultry trade routes between major population centres in these regions [58] or to trade routes along the Mekong River from Lao PDR to Vietnam. Some viruses may have spread back and forth between countries at different time points [48]. Cross-border poultry trade between Vietnam and China could have led to the introduction of clade 2.3.4 (presumably from Guangxi province, China) and other lineages into Vietnam [48].

The H5N1 virus became widespread and endemic in Thailand throughout 2004 without interruption, despite brief periods of undetectable transmission during the hottest months of February–May [29]. It is believed that the virus is maintained in Thailand at low level during these dry, hot months, therefore providing a source for outbreaks during the wet and cooler months of September–December, when the conditions are more favourable for its spread. A new viral strain (clade 2.3.4, genotype V) was introduced into the north-eastern region of the country in 2006 [50]. The subtype H5N1 viruses circulating in the markets in ten provinces of central Thailand during July 2006–August 2007 were genetically related to those that circulated in Thailand during 2004–2005, which indicated that the virus was endemic to Thailand [62]. This suggests that yearly re-emerging viruses in central Thailand belonged to a similar lineage and that they originated from a locally persistent reservoir, rather than repeated introductions [30,63]. Recent avian influenza outbreaks in Thailand in 2007–2008 were also shown to be caused by indigenous viruses [33].

In Cambodia, the HPAI H5N1 virus was probably introduced from Thailand in 2004 [64] and then re-introduced from Vietnam over several waves of transmission until the virus finally became endemic, establishing a recent sub-lineage in the south Indochina peninsula region. Poultry movements, rather than repeated re-introductions of H5N1 viruses by wild birds, are responsible for virus circulation and perpetuation [64]. Within Cambodia, the spread of H5N1 virus clearly occurs in a North-to-South direction, following a major road and transport route. Poultry trading, live poultry markets, cock fights, and other risk factors for poultry contamination are therefore likely to be responsible for the spread of the virus [65,66].

In Lao PDR, it seems that the H5N1 virus has not persisted endemically. H5N1 was first detected in 2003, and then disappeared in 2004 [38]. In early 2006, a clade 2.3.4 virus was detected, which was presumably introduced from Northern Vietnam [38]. This virus also seems to have vanished from Lao PDR [38]. However, in 2007, a genotype V, clade 2.3.4 strain was isolated near the Thai border. Phylogenetic analysis of the HA gene showed that this strain probably shared a common origin with Thai strains isolated in 2006 near the border. Although the direction of the transmission can not be determined, extensive poultry movement across the Mekong River is believed to have facilitated the circulation of these strains between countries [51].

In Indonesia, phylogenetic analysis suggests that a single introduction of genotype Z, clade 2 viruses from Southern Chinese domestic poultry (Hunan province) has occurred (Figure 4) [4,47,67]. The continuing endemicity of those viruses subsequently resulted in the establishment of geographically distinct groups [8]. In the Indonesian sublineages, there are three groups of viruses. The first group includes viruses from Central and Eastern Indonesia (Java, Southern Sulawesi and West Timor). The second group also contains viruses from Central and Eastern Indonesia (isolates from Java, Bali, Flores Island and West Timor). In comparison, the third group of viruses are from Central and Western Indonesia, found throughout Java and Sumatra, and Bangka Island [8]. These relationships highlight the subsequent spread both east and west throughout the country. Continued virus activities in Indonesia were attributed to transmission via poultry movement within the country rather than through repeated introductions by bird migration [8]. Java is thought to be an epicentre for H5N1 generation and spread in Indonesia [8,68] (Figure 4).

## Evolution of Influenza A(H5N1) Virus Genes

### Hemagglutinin (HA) gene

Diverse populations of endemic HPAI H5N1 viruses have continuously evolved in South East Asia, from 2003 until the present day, and several gene modifications have occurred within these viruses which may affect their transmissibility and pathogenicity. To become a pandemic strain, a H5N1 virus must be able to be efficiently transmitted between human hosts, a feature that existing H5N1 viruses have not yet acquired.

The HA is the main influenza antigen and determines cell binding, host range, and neutralizing antibody response. Thus, some modifications on the HA gene could provide the virus with advantageous properties. Several key amino acid residues on the HA have been identified.

Avian influenza viruses are defined as HPAI or LPAI viruses, based on the severity of the disease which they cause. HPAI viruses are highly contagious amongst poultry, and often result in a high fatality rate, especially in terrestrial poultry like chicken, quail or turkey (up to 100% in 2–3 days). LPAI viruses are responsible for mild diseases, with few or no symptoms in some bird species. The defining feature of HPAI is a multi-basic amino acid motif at the cleavage site present in the HA gene (PQRERRRKKR/G), which confers increased pathogenicity. LPAI viruses have a single arginine at the HA cleavage site, which can subsequently be cleaved only by trypsine-like proteases. Since these proteases are present in a restricted number of organs, the infection is usually limited to the respiratory or to the intestinal tract, without becoming systemic. In contrast, the presence of multiple basic amino acids allows the hemagglutinin to be cleaved by many different proteases, enabling a broader tissue tropism, and the ability to cause systemic infections. Some H5N1 strains have alterations on this site, such as Arg (R) or Lys (K) deletions or insertions. Alterations of this kind were observed, among others, on Cambodian, Indonesian, Thai and Vietnamese H5N1 isolates [8,49,50]. Although the real impact of such changes in the HA cleavage site is difficult to estimate, the high frequency of these occurrences highlights the importance of conscientious surveillance of this site.

The relevant residues involved in antigenic sites on the H5 HA have been described, and compared to H3 antigenic sites which have been comprehensively characterized [69,70]. The host immune pressure can induce mutations on these antigenic sites, which results in the emergence of immune-escape mutants. Therefore, a positive selective pressure on those sites can rapidly lead to ineffective host immune responses, which are a major constraint for the development of human or animal vaccines. In Vietnam and Indonesia, among virus isolated from August 2003 to June 2005, a positive selective pressure has been reported on 8 residues of the HA gene. Five of those residues were located on antigenic sites A and E (positions 83, 86, 138, 140 and 141), two of them were suggested to be involved in receptor binding (positions 129 and 175). The last one was a potential *N*-linked glycosylation site (position 156) [8].

The HA of human influenza viruses preferentially bind to sialic acid (SA) receptors linked to galactose (Gal) through an α-2,6 linkage (SA α-2,6Gal), whereas avian influenza viruses preferentially bind to SA receptors of the α-2,3 linkage (SA α-2,3Gal). The HA sites which bind these receptors are often under selective evolutionary pressure. Mutations of these binding sites from an avian to human receptor binding preference could facilitate human-to-human transmission and these sites have been proposed as markers for assessing the pandemic potential of H5N1 isolates [71]. Two key residues have been identified to determine receptor preference switching – position 226 and 228 in H2 and H3 isolates, which corresponds to 222 and 224 in the H5 gene [72]. However, the mutations Gln222Leu and Gly224Ser which are believed to enhance the affinity of the virus for the SA α-2,6Gal have never been observed in H5N1 field isolates. Nevertheless, several naturally occurring mutations were associated with an enhanced affinity of the avian virus to “human type” receptors SA α-2,6Gal. Mutations at position 182 (Asn182Lys) and 192 (Gln192Arg) can independently switch the receptor binding preference from avian to human [73]. Several other combinations of mutations were found to have a variable effect on receptor switching, certainly indicating that in the right circumstance a virus may adapt to bind human receptors more efficiently.

H5N1 HPAI viruses from clade 2.1 (found in Indonesia) may be under a lower positive selective pressure compared to the other clades [74]. Although such an observation is difficult to interpret, there may be a reduced necessity for the virus to evolve and adapt in Indonesia due to the high endemicity of the disease. The persistence in poultry reduces the need for the virus to jump from one species to another, especially to mammalian species, in order to maintain the chain of transmission [74].

### Neuraminidase (NA) gene

The neuraminidase enzyme cleaves HA from sialic acids, facilitating the release of newly assembled viral particles from the host cell surface and thus enabling spread to other cells. Interestingly, all H5N1 viruses which have been isolated post-2003 contain a deletion in the stalk region of the protein, at positions 49–68 for clade 1 and 2 viruses, and at positions 54–72 for clade 3 viruses [75]. This deletion reduces the enzymatic activity of the NA, and may act to balance with the HA which has a reduced affinity interaction with receptors in poultry, compared to aquatic birds [76].

The NA is a target for the neuraminidase inhibitor (NAI) class of antiviral drugs, including Oseltamivir (Tamiflu™) and Zanamivir (Relenza™) which are commercially available. H5N1 viruses are typically sensitive to NAI’s, and these drugs are used as first line treatment for H5N1 infections in humans, and are a major component of pandemic planning as they are also recommended for chemoprophylaxis [77]. The His274Tyr mutation is associated with resistance to Oseltamivir. This mutation is now increasingly prevalent in seasonal H1N1 and H3N2 influenza strains, partly due to widespread use of Oseltamivir [77]. In addition to His274Tyr, amino acid substitutions Arg292Lys, Glu119Val and Asn294Ser have been associated with resistance or reduced sensitivity to Oseltamivir [78].

There have been several reports illustrating the development of Oseltamivir resistance during the treatment of H5N1-infected patients. Some patients developed the His274Tyr mutation, others the Asn294Ser mutation associated with a decreased sensitivity to NAI’s [79,80]. To assess the threat of a resistant H5N1 strain persisting with equal fitness and replicability, the H5N1 virus A/Vietnam/1203/2004 was engineered to possess several NA resistance mutations including His274Tyr, Glu119Val, Arg292Lys, and Asn294Ser. Viruses with His274Tyr and Asn294Ser were found to retain their infectivity and pathogenicity, highlighting the importance of ongoing surveillance of Oseltamivir resistance for early detection of resistant viruses [81].

In the absence of resistance mutations, genetic drift mutations also give rise to variations in Oseltamivir sensitivity [82,83]. Mutations which are remote from the active site may not confer absolute resistance, but may affect the dosage of drugs required to treat these infections. Suboptimal dosing may lead to the emergence of fully resistant viruses. Clade 2.3.4 viruses in Vietnam were recently found to be 8-fold less susceptible to Oseltamivir, but maintain their susceptibility to the adamantanes (M2 inhibitor drugs) [56]. Treatment combining NAI and adamantanes is recommended for patients infected with adamantane-sensitive H5N1 strains [56,84]. Ongoing evaluation of the evolution of drug sensitivity in both seasonal and H5N1 influenza viruses is vital in understanding the best treatment options in the wake of rapid virus evolution.

### M gene

The M gene encodes two capsid proteins (M1 and M2). M1 is a RNA-binding protein, and M2 is a membrane ion channel protein involved in H^+^ proton transport, which facilitates the acidification of the endosome and the release of the viral particle inside the cytoplasm [85]. A comparison of synonymous and non-synonymous nucleotide substitutions in virus isolates from Indonesia and Vietnam (2003–2005) provided evidence of positive natural selection on the M2 gene [8]. Mutations on the M2 protein can result in different adaptative processes which affect the virus’ capacity to uncoat during the early stages of the infection, and modulate its virulence. Also, mutation on the M2 gene can lead to adaptation to the different pH environments of aquatic or terrestrial hosts [8]. Moreover, the M2 protein is the target of the adamantane class of antiviral drugs (amantadine, rimantadine). Two mutations on this protein (Leu26Ile and Ser31Asn) lead to drug resistance, and these mutations are present in all genotype Z, clade 1 viruses [86]. Clade 2.3.4 viruses from Indonesia and Vietnam remain sensitive to amantadine [56], and clade 2.1 and 2.2 viruses have varying rates of resistance to this class of drug [86,87]. Thus, monitoring M2 mutations in H5N1 viruses remains an important aspect of avian influenza surveillance.

### Polymerase complex (PB1, PB2, PA)

The polymerase complex of influenza viruses is composed of three subunits: PB1, PB2, and PA. These subunits, along with the NP protein and the RNA genome, form the ribo-nucleoproteic complex, necessary for the protection of the genome, as well as the replication and transcription processes. The PB1 gene also encodes the PB1-F2 protein by an alternative reading frame. Analyses conducted on viruses from Vietnam and Indonesia isolated between 2003 to 2005 showed that several sites on the PB1-F2 gene were under positive natural selection, although the significance of these particular sites is not known [8]. In mouse models and in cell lines, the PB1-F2 protein can influence viral pathogenicity, by sensitizing host cells to apoptotic stimuli (e.g. TNFα), thereby promoting apoptosis during infection [88,89]. However, the residues involved in this process have not yet been identified.

Mutations at residue 627 on the PB2 protein are associated with increased virulence and are thought to be relevant to the adaptation of H5N1 viruses to human hosts. The 627 residue on PB2 is a glutamic acid (Glu) in avian strains [76]. The Glu627Lys mutation on the PB2 improves replication efficiency, enhances adaptation of HPAI viruses to mammalian hosts, and increases transmission between mammalian hosts in experimental models [90–92]. The 627Lys mutation enhances growth at lower temperatures, consistent with those found in the human upper respiratory tract (around 33°C), compared to wild type viruses with 627Glu which grow optimally at 41°C [93,94]. Within clade 1 or clade 2.1 viruses, the Glu627Lys mutation is observed in some human strains but not in avian strains, suggesting a selective advantage for these isolates in mammalian hosts [8,95]. In clade 2.2 viruses, the Glu627Lys mutation is observed in both human and avian strains [47]. Interestingly, most of the strains isolated in birds from the Qinghai Lake outbreaks in 2005 (clade 2.2), which spread to Europe and Africa, contained the 627Lys mutation [60,96].

In addition to residue 627, several other changes on the PB2 protein and other proteins of the polymerase complex also contribute to adaptation to mammalian hosts and to regulation of virulence [90,97]. Positive selective pressure was also detected at several sites of unknown function, on PB2 and PA genes from Indonesian strains isolated from 2003 to 2007 [39]. Thus, surveillance of the substitutions occurring on the polymerase genes, and further elucidation of the importance of PB2 and PA genes in virus evolution is of ongoing interest.

### NS1 protein

The non-structural protein NS1 can regulate H5N1 virus virulence in humans by modulating the host immune response. The severity of human infections with H5N1 virus in 1997 was partly due to the resistance of these viruses to interferons and TNFα during the host immune response. The presence of glutamic acid at residue 92 on the NS1 was vital for these effects [98,99]. However 92Glu has not been identified in more recent human H5N1 strains. Ser42 and Ala149 can also inhibit immune response signaling pathways, including induction of interferons [100,101]. On the contrary, deletions in the NS1 gene can attenuate virulence [102]. The NS1 carboxy-terminal PDZ ligand binding motif is also a potential virulence factor. This motif binds cellular PDZ-containing proteins, disrupting a range of cellular signaling pathways [103]. Therefore, monitoring the evolution of the NS1 gene is important for the detection of novel viruses with increased pathogenicity in humans.

## H5N1 virus host range

The H5N1 avian influenza virus does not easily infect humans and cannot spread efficiently amongst the human population. Very few suspected cases of human-to-human transmission have been suspected in family clusters of patients, which account for approximately one quarter of total number of cases [40,104]. In most cases, patients probably acquired infection from common-source exposures to poultry or contaminated environment. In some cases, family members in close and unprotected contact with severely ill patient have become ill in the apparent absence of other exposure factors [39,104,105]. Several studies have demonstrated a lack of human-to-human transmission in high-risk populations such as unprotected health care workers [106,107], therefore it remains unclear whether successful human-to-human transmission has occurred in select cases.

It is noteworthy that more and more cases of avian to mammalian infections with H5N1 virus did naturally occur or were experimentally possible [108,109]. In Indonesia there is a seroprevalence of H5 neutralizing antibodies of up to 20% in the cat population living near poultry markets were H5N1 virus has been circulating [110]. There are also reports in other carnivores like dogs, tigers and leopards, suggesting that H5N1 virus has a potentially broad host range [111,112].

## Conclusions

The H5N1 virus which originated in Southern China has spread across 3 continents and is now endemic in many South East Asian countries, with far-reaching effects on public health and local economies. Vietnam and Indonesia have experienced the greatest number of H5N1 outbreaks in both poultry and humans. The Gs/Gd lineage has evolved into multiple novel genotypes through reassortment. Clades are continuously diversifying and requiring further sub-classification. The transduction of H5N1 across South East Asia has occurred through poultry movements and migratory birds. Molecular characterization of H5N1 viruses is an ongoing priority, essential for monitoring antigenic changes, antiviral sensitivity and pathogenicity of novel strains. The pandemic potential of H5N1 virus, 12 years on from its original detection, should not be underestimated.

## Figures and Tables

**Table 1.** Summary of H5N1 outbreaks in poultry and humans in South East Asia.

**Country**	**Year of first outbreak**	**Number of outbreaks in poultry**	**Number of human infections**	**Number of human deaths**	**Country Fatality Rate**
**Cambodia**	2003	21	8	7	88%
**China**	1996	98	38	25	66%
**Indonesia**	2003	261	141	115	82%
**Lao PDR**	2003	18	2	2	100%
**Malaysia**	2004	16	0	0	-
**Myanmar**	2006	93	1	0	0%
**Thailand**	2004	1141	25	17	68%
**Vietnam**	2003	2539	111	56	50%
